# Controlling for conservation in genome-wide DNA methylation studies

**DOI:** 10.1186/s12864-015-1604-3

**Published:** 2015-05-30

**Authors:** Meromit Singer, Lior Pachter

**Affiliations:** Division of Computer Science, University of California at Berkeley, 94720 Berkeley, CA USA; Current address: Broad Institute of MIT and Harvard, 415 Main Street, 02142 Cambridge, MA USA; Department of Mathematics, University of California at Berkeley, 94720 Berkeley, CA USA; Department of Molecular and Cell Biology, University of California at Berkeley, 94720 Berkeley, CA USA

**Keywords:** Averaging, Conservation, Comparative analysis, Missing data, DNA methylation, Junctions, Intron, Exon, Coding

## Abstract

**Background:**

A commonplace analysis in high-throughput DNA methylation studies is the comparison of methylation extent between different functional regions, computed by averaging methylation states within region types and then comparing averages between regions. For example, it has been reported that methylation is more prevalent in coding regions as compared to their neighboring introns or UTRs, leading to hypotheses about novel forms of epigenetic regulation.

**Results:**

We have identified and characterized a bias present in these seemingly straightforward comparisons that results in the false detection of differences in methylation intensities across region types. This bias arises due to differences in conservation rates, rather than methylation rates, and is broadly present in the published literature. When controlling for conservation at coding start sites the differences in DNA methylation rates disappear. Moreover, a re-evaluation of methylation rates at intronexon junctions reveals that the magnitude of previously reported differences is greatly exaggerated. We introduce two correction methods to address this bias, an inferencebased matrix completion algorithm and an averaging approach, tailored to address different underlying biological questions. We evaluate how analysis using these corrections affects the detection of differences in DNA methylation across functional boundaries.

**Conclusions:**

We report here on a bias in DNA methylation comparative studies that originates in conservation rate differences and manifests itself in the false discovery of differences in DNA methylation intensities and their extents. We have characterized this bias and its broad implications, and show how to control for it so as to enable the study of a variety of biological questions.

**Electronic supplementary material:**

The online version of this article (doi:10.1186/s12864-015-1604-3) contains supplementary material, which is available to authorized users.

## Background

**Fig. 1:**
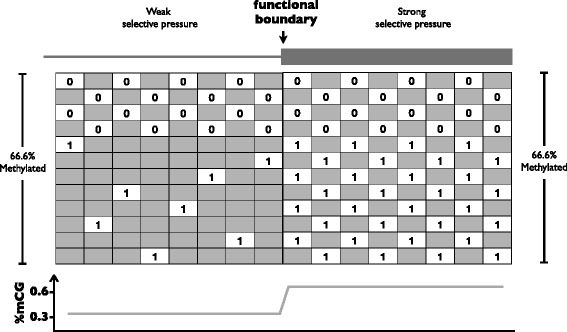
Bias introduced by the Yule-Simpson effect. A comparative analysis of DNA methylation across a functional boundary. A pair of matrices is associated to a functional boundary (one for each side of the boundary). The methylation states are represented by numeric values at all relevant genomic locations (here 0 and 1 are used for simplicity, but any values may be used). For example, row five may represent a genomic locus: CGAGTCAG||CGCGCGCG (|| marking the functional boundary location) where all CG sites are fully methylated. The mean of the row averages (*R*) is 0.67 for both matrices: in eight of the 12 regions all CGs are completely methylated, and in the remaining four regions all CGs are completely unmethylated. The mean of the column averages (*C*) is 0.33 for the *left matrix* and 0.67 for the *right matrix*. Although there is no difference in the regional methylation average across the matrices, a difference in the column averages is observed due to a different data distribution: regions that are methylated are represented with fewer CGs in the *left matrix* than they are in the *right matrix*, but this is not the case for unmethylated regions. Importantly, it is not the lower frequency of sites in the *left matrix* that causes the discrepancy between *C* and *R* but the lower frequency of sites in the methylated regions

High-throughput molecular biology is enabling the molecular characterization of functional regions by aggregation of genome-wide data. Examples include the assignment of DNA methylation intensities to functional regions (e.g. exons) by averaging over the DNA methylation state of many single instances, or the assignment of conservation scores to genomic locations by aggregation of the information in multiple sequence alignments. The latter analysis was first performed in the mouse genome paper (Fig. 25a, [1]) and has recently been highlighted as being of historic significance [2]. The averaging of signal across multiple regions of the same functional type facilitates the reduction of genome-wide results to summary statistics, but is complicated by the presence of missing data, e.g. missing CpGs in the case of methylation or indels in the case of alignment.

In the case of DNA methylation, many studies use the aggregation of methylation states at multiple CpG (CG) sites across many regions to compensate for the sparsity of signal at any specific location relative to a boundary. Such analyses, where the percentage of methylated cytosines (%mCG) is computed at each location relative to a boundary (Fig. 1), are currently considered standard when characterizing the methylomes of new species [3–18], and are frequently used to compare methylation intensities at functional boundaries such as intron-exon and UTR-coding junctions [19–29].

In this paper we address the validity of such comparisons. We show that the %mCG rates at the different relative locations cannot be directly used to deduce the methylation states of regions, or to determine whether different functional regions exhibit significant differential methylation (Fig. 1). We explain that this is a byproduct of selection, which leads to an association between the number of CGs in a specific region and the extent to which the region is methylated. Specifically, deamination of methylated cytosines results in depletion over time of CGs in constitutively methylated regions of vertebrate genomes [30]. Varying evolutionary constraints affect the pattern of CGs, thereby limiting the availability of methylation data in some regions. The non-uniform distribution of “missing data” (absence of CG sites), associated with varying selection rates, results in the Yule-Simpson effect (or Simpson’s paradox) [31–36] confounding the naïve analyses (Fig. 1 and Additional file 1: Fig. S1). As explained in more detail in the next sections, the Yule-Simpson effect concerns the possibility that average-based comparison studies over aggregations of instances may lead to different conclusions than comparisons conducted without aggregation, due to an underlying trend of varying data distributions. Additionally, we show that this bias is present also when relative changes are considered, e.g., when comparing the relative change in DNA methylation under several different experimental conditions. We have found that numerous DNA methylation studies may be vulnerable to these issues, and show this to be the case for human methylome analysis.

The biases prevalent in current studies must be addressed by modifying the statistical analyses conducted. We introduce two methods for this task that are suitable for many genome-wide studies where signal is aggregated by functional region. The first method involves regional averaging on a reduced dataset. While it is robust and involves minimal statistical inference, this method requires ignoring a significant portion of the collected data, and cannot determine quantitative and site-specific methylation tendencies. The second method we propose applies a model-based matrix completion algorithm to circumvent the missing data problem. It is inspired by machine learning techniques for matrix completion applied in other settings [37], and in our case we are able to take advantage of specific patterns linking conservation pressure to a predicted observed state. The method learns the relevant set of parameters separately for each dataset, and generates site-specific inferences of the measure of interest, along with comparative statistics and error estimates.

Using our approaches, we find that previous studies exaggerate the difference in methylation between functional regions, and our analysis of previously published data leads to different conclusions about the functional role of DNA methylation. Finally, we discuss additional settings in which this bias can be present and introduce a method to test for its presence in arbitrary datasets.

## Results

### Averaging-based comparisons of DNA methylation intensities are biased by uneven distribution of data

The bin- or site-specific approach to DNA methylation comparisons across region types can be formalized as follows (Fig. 1): The methylation states on each side of a functional boundary are represented by matrices. Each genomic region of interest is split at the junction, with each side contributing a row to the relevant matrix. Each column represents a distance from the boundary (this can be site-specific or relative if using binning). The *ij*-th entry of a matrix indicates the extent to which region *i* was methylated at distance *j* from the boundary. In the case that the base at location *j* in region *i* cannot be methylated (for example, it is not a cytosine or part of a CG site), the matrix contains “missing data” (grey boxes in Fig. 1).

To illustrate the difficulties in comparing methylation across boundaries, we show in Fig. 1 a simplified hypothetical example in which regions across a functional boundary have the same methylation tendency. In this case the CG sites on both sides of a junction are either all methylated or all unmethylated. However a naïve comparison of the column averages suggests a significant increase in the average mCG/CG rate when crossing the functional boundary. This apparent “paradox”, can be understood by noting that the increase observed in the column averages is a result of the missing data being differently distributed across the functional boundary: regions upstream of the boundary have less data points at methylated regions than the coupled downstream regions, but there is no such trend for unmethylated regions. The “step” in column averages observed in the example is due to the different tendencies for missing data across the boundary. We emphasize that it is not the difference in the *number* of data points that results in an observed difference in the mCG/CG measure, but the difference in the number of data points *specifically at the methylated regions*. If all rows on the left hand side of the junction had one CG, a significant difference would not be observed. In the case of Fig. 1, comparison of the row averages would reveal that there is no difference in methylation tendency across the functional boundary. However, as we will show in the next section, row averages may also be inadequate for comparison of methylation, particularly in the common case that some rows do not have any CGs.

We hypothesized that the effects highlighted in Fig. 1 arise in genome-wide methylation analyses due to the process of deamination, which results in the loss of CG sites at regions that are constitutively methylated (or methylated in the germline). While the process of deamination leads to the loss of CG sites in methylated regions under weak selection, regions under strong selection maintain more CG sites, regardless of their methylation state. This suggests the presence of several different biases in current comparative studies. Importantly, it implies that “column-based” methylation comparisons are confounded by the extent to which regions vary in selection. We begin by describing the extent of bias that can be introduced by averaging, and follow that with a demonstration of the extent of the problem using whole genome methylation data.

#### Site specific averaging is not equivalent to regional averaging

We define an *nxm* aggregation matrix ***W*** as follows: each row of ***W*** represents one of a set of genomic regions of equal length. At each row, locations of the corresponding region that do not have CG sites^1^^1^ correspond to empty elements in the matrix, and are referred to as “missing entries” or “missing data”. Sites that can be methylated are represented with the assigned methylated values; in the case of bisulfite sequencing, values between 0 and 1 (Fig. 1). Given an aggregation matrix ***W***, we define *M*_*W*_ to be the mean over all elements of the matrix, *R*_*W*_ to be the mean of the row averages and *C*_*W*_ to be the mean of the column averages. For example, if we set ***W*** to be the matrix on the left in Fig. 1, we have *M*_*W*_ = *1*/*3*, *R*_*W*_ = *2*/*3* and *C*_*W*_ = *1*/*3*. For the case in which ***W*** has no missing values, it is easy to see that *M*_*W*_ = *R*_*W*_ = *C*_*W*_. However, the following proposition establishes that matrices with missing values can have (arbitrarily) different *M*_*W*_, *R*_*W*_ and *C*_*W*_ values (see proof in Additional file 2: Supplementary text).

##### Proposition 1.

The difference between column averages and row averages can be arbitrarily large; for any three rational values *r*, *c* and *m* (*0* < *r*,*c*,*m* < *1*), there exists an aggregation matrix ***W*** such that *R*_*W*_ = *r*, *C*_*W*_ = *c* and *M*_*W*_ = *m*.

We tested the extent to which these measures differ for different types of regions in the human methylome (Table 1). Establishing the extent to which these measures differ in analyzed datasets is important because in previous work *C*_*W*_ [3–29, 38–41], *R*_*W*_ [6, 7, 14, 16, 26, 42, 43] or *M*_*W*_ [19, 20, 43–47] were used in comparisons of methylation across region types, with *C*_*W*_ being the prevalent choice. Table 1 shows that the difference between *C*_*W*,_*R*_*W*_ and *M*_*W*_ is large in some cases, and that the magnitude of this difference varies between regions of different types. For example, in promoter regions there is a difference of 0.1 between *R* and *M*, whereas in intronic regions there is a difference of 0.04 between *C* and *R*, approximately half of the reported difference in methylation between introns and exons at junctions [19, 24]. We observed that in general the extent of discrepancy between the measures is inversely associated with the extent of selection. Specifically, exons show the smallest differences.

**Table 1:** Statistics for averaging-based analysis of the human methylome

	C	R	M	r
Promoters	0.19	0.28	0.18	−0.50
Introns	0.69	0.73	0.69	−0.14
Exons	0.79	0.78	0.79	0.04
Enhancers	0.33	0.36	0.33	−0.14
p53 binding sites	0.07	0.13	0.1	−0.18

The means of column averages (*C*), row averages (*R*) and all matrix values (*M*) are shown for different region types (Methods). Also listed are the Pearson correlations (r) between the number of sites in each row and its mean methylation.

We hypothesized that the differences between measurements are due to a negative correlation between the extent to which a region is methylated and the number of CG sites. We reasoned that such a process would affect *C*, *R* and *M*, but that *C* and *M* would be affected to a stronger extent than *R*. Indeed, we observed such negative correlations for all but the exon regions (Table 1). At coding exons, such associations are not observed, probably due to strong selection preventing the loss of CG sites in methylated regions.

#### Average-based comparisons across region types can lead to exaggerated difference estimates and false findings

Having established that different average-based measures are not equivalent to each other, and that they do not necessarily capture the actual methylation tendency, we proceeded to investigate how the use of column, row or matrix averages affects comparative methylation studies.

We found that a direct implication of possible differences between average-based measures is that a comparison of the DNA methylation between two types of regions (e.g., intronic and exonic regions at intron-exon boundaries) is not the same as a comparison of the site-averages of methylation states. This is known as the “Yule-Simpson effect” (YS) [31–35], and it follows from the following corollary to Proposition 1: two matrices, ***W***_***1***_ and ***W***_***2***_, can have the property that *C*_*1*_–*C*_*2*_ is different from *R*_*1*_–*R*_*2*_. The direct relationship between our setting and a classic example of the YS effect is detailed in Additional file 1: Fig. S1.

Usually the term “Simpson’s paradox” refers specifically to a reversal of effect when using averages. In our terminology this is equivalent to the statement that for matrices ***W***_***1***_,***W***_***2***_, we have that *R*_*W1*_ > *R*_*W2*_ while *M*_*W1*_ < *M*_*W2*_. The following proposition connects this discord directly to the classic Simpson’s paradox (see proof in Additional file 2: Supplementary text, and an example of occurrence at an intron-exon subset in Additional file 1: Fig. S2).

##### Proposition 2.

The sign of column-average and row-average comparisons across regions may be inconsistent. Let ***W***_***1***_ and ***W***_***2***_ be two aggregation matrices. *R*_*W1*_–*R*_*W2*_ can have opposite sign to *C*_*W1*_–*C*_*W2*_, and if it does then an instance of Simpson’s paradox has occurred.

**Fig. 2:**
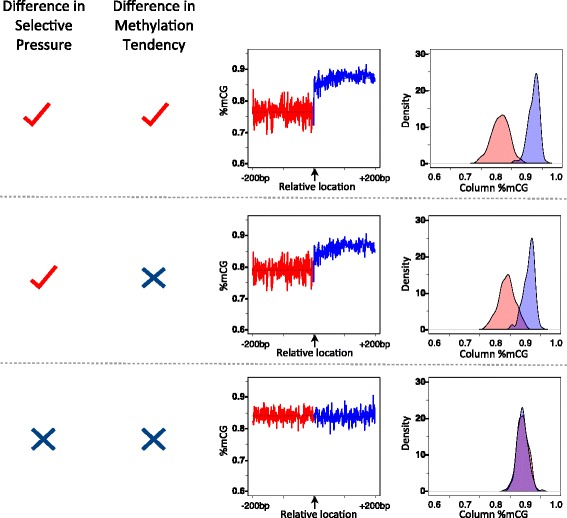
An observed difference in the mCG/CG rate across a functional boundary is due to a change in conservation rates. A simulation based on the genetic structure of intron-exon junctions of the human genome (maintaining the location of CG sites). *Top row*: maintaining the original genetic structure (location of CG sites), and simulating a difference of 3.2 % in methylation tendency (Methods). *Middle row*: maintaining the original genetic structure (location of CG sites), and simulating no difference in methylation by shuffling the methylation values across each matrix row (Methods). *Bottom row*: simulating no difference in selective pressure or methylation tendency by shuffling all values (both missing and CGs) at each matrix row. Shown are the %mCG/CG site-specific values and histograms of all %mCG/CG values for the different region types

**Fig. 3:**
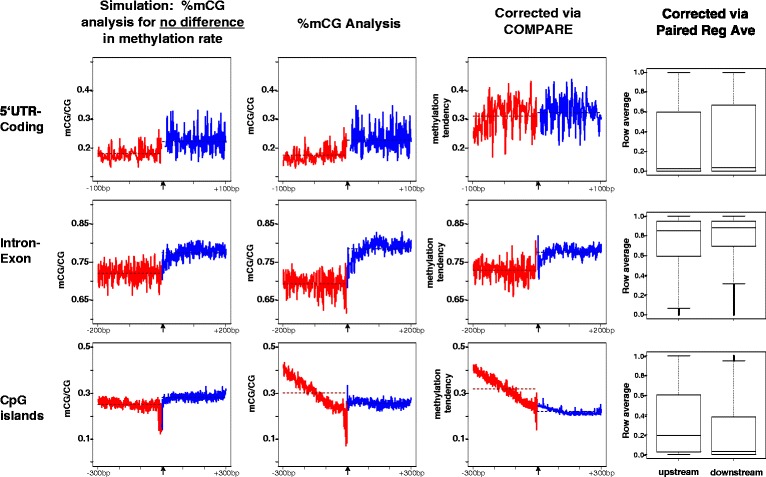
Analysis of DNA methylation at functional boundaries. Matrices containing bisulfite-generated methylation estimates were constructed from the relevant instances of the human genome (hg18), incorporating 4852 5′UTR-coding junctions, 20,784 mid-gene intron-exon junctions and 21,408 CG island junctions. Each plot shows average methylation measurements at increasing distances relative to the junction (different columns of the matrix). The means of the column-average values are shown as dashed lines. Simulation (*leftmost column*)- the methylation values for each pair of coupled matrix rows were shuffled according to a random permutation (maintaining the locations of missing values), resulting in simulated data with equal methylation tendency across the junction. Differences observed in the mCG/CG measures are due to differences in the matrix structure across the boundary (as in Fig. 1). %mCG Analysis (*middle*)–naïve averaging of the values in the matrix columns. Corrected (*rightmost columns*)–column-averages after inference with COMPARE and comparisons after correction with Paired Region Averaging

To test the extent to which comparative studies of the human methylome are confounded by the YS effect, we conducted simulations in which the methylation tendencies were set to be equal for the regions compared, while the matrix data structure, i.e., locations of CG sites, was kept the same (Methods). We found that although, as expected, there was no true difference in the tendency of CGs to be methylated, a “step” was observed when comparing the column averages (Figs. 2 and 3 leftmost panel). The presence and size of the step in these simulations indicate the need to correct for the YS effect to attain accurate comparisons of methylation tendencies across regions, that are not confounded by a difference in selection rates. An additional example of the need to correct for confounding selection effects is apparent when considering a subset of intron-exon junctions for which the intronic regions have higher methylation average rates than the downstream exonic regions (Additional file 1: Fig. S2). In this case *C*_*Intron*_ is significantly smaller than *C*_*Exon*_ (*C*_*Intron*_ = 0.75, *C*_*Exon*_ = 0.78, *p* < 2.2e-16), but *R*_*Intron*_ is significantly larger than *R*_*Exon*_ (*R*_*Intron*_ = 0.82, *R*_*Exon*_ = 0.78, *p* < 2.2e-16).

Investigating further, we found that in some cases reported differences in methylation seem to be due to the YS effect, rather than a difference in the methylation tendency of the regions at hand. 5′UTR-Coding junctions have been reported to display a significant difference in methylation rate based on a difference in the mCG/CG value distribution (which corresponds to comparing *C* values across the matrices) [9, 26, 28]. We observed such a significant difference (0.05, *p* < 2.2e-16) in a replicated analysis using whole-genome bisulfite data (Fig. 3, upper panel). However, when considering regional averages in a coupled corrected manner (Methods and next section) the difference drops to 0.02, and is non-significant (*p* = 0.08, Mann–Whitney-Wilcoxon test). Additionally, in a simulation shuffling methylation values such that there is no difference in methylation tendency across the junctions, a significant difference remains when conducting *C*-based analysis (Fig. 3, top-left plot). The difference observed in simulation is due only to the presence of the YS effect as described. We therefore conclude that there is no significant difference in methylation tendency and extent at 5′UTR-Coding junctions in the human methylome.

The YS effect is not just a feature of analysis at site-specific resolution. It can also lead to questionable conclusions in bin-based studies. For instance, it has recently been reported that there is a significant difference in 5-hydroxymethylcytosine (5-hmC) across the exon-intron boundary in the human brain [23], and that the change in 5-hmC was most evident at a 5 bps distance from the boundary (*p* = 2.3 × 10 − 6). However, a closer look reveals that although 48,564 exon-intron junctions were present in the dataset analyzed, only 22 and 501 have methylation values at the 5 bp region from the boundary, at the intron and exon side, respectively. Moreover, there is not a single instance at which there were 5-hmC values on both sides of the boundary. As we have reported, the difference in selection between introns and exons results in a different distribution of missing data between these regions. We therefore conclude that there is currently no evidence for a significant difference in methylation or hmC rate across the exon-intron junctions at 5 bps resolution. Testing for the possibility of such a difference at such fine resolution would require adding statistical power, perhaps by integrating more chromosomes and additional species into the analysis.

### Correction methods allow for comparisons of DNA methylation tendencies across regions of different conservation rates

**Fig. 4:**
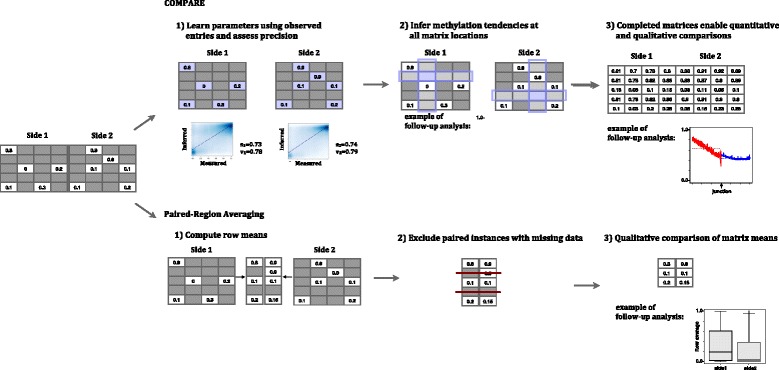
The COMPARE and Paired-Region Averaging methods for correcting biases introduced by differences in conservation rates. *Top*: COMPARE learns a different set of parameters for every column, and the methylation tendency of site *i*,*j* is inferred from the features of row *i* and the parameters set for column *j*. The complete inferred matrix can then be used in subsequent average-based tests. *Bottom*: Paired-Region Averaging computes for each instance (e.g. a specific intron-exon junction) the average methylation rate at each side of the functional boundary and excludes instances at which either of the boundary sides is lacking any values (and hence no average-based DNA methylation rate can be assigned). Tests to compare the relative methylation tendencies of the different boundary sides can be applied, but a quantitative analysis of the DNA methylation rates cannot be conducted when using this method due to the region-exclusion step

In this section we introduce two methods for unbiased comparisons in settings in which the YS effect we report on is present, and apply them to the comparison of DNA methylation tendencies (Fig. 4). We describe the methods in the context of DNA methylation comparisons, but note that the techniques and software described can be extended to apply to different settings in which the YS effect is present (see Discussion). We first describe each method and then present results from their application to instances of the human methylome.

#### Paired region averaging

In order to account for the YS effect, regional averages may be leveraged to compare methylation tendencies across functional boundaries. However, comparisons using regional averages require adjustments to avoid biases. In the correction we propose, the regional average (row average) of methylation tendencies is computed separately for the two sides of each boundary instance, discarding from subsequent analysis boundary-instances at which one or both sides of the boundary do not have any measured methylation values (Fig. 4). The methylation tendency at the different sides of the boundary can then be compared using the statistical test of choice (Fig. 3, rightmost column).

While allowing for comparisons of methylation tendencies in an unbiased manner, the paired row-averaging approach has several shortcomings. First, it requires discarding data. While this is crucial to avoid the YS effect (see Fig. 4 and Additional file 1: Fig. S3), due to the sparsity of CG sites at methylated regions a considerable amount of information may need to be discarded. For example, when considering intron-exon junctions at 200 bp resolution, 6253 of 20,784 intron-exon junctions are discarded, and the number grows for shorter distances from the boundary. Second, while this method enables an unbiased assessment of the relative methylation tendency across functional boundaries (determining if the methylation tendency on one side of the boundary is larger than the other), it does not give a quantitative measurement of the methylation tendencies (Additional file 1: Fig. S3). This follows from the fact that instances of missing data are biased to originate from more highly methylated regions. Third, the regional averages of methylation tendencies do not allow for site-specific assessment and therefore cannot determine if changes in methylation tendencies occur at a gradual or step-wise manner.

Nevertheless, paired row averaging is a sound method for the comparison of DNA methylation tendencies across region types, and does not require statistical inference. Figure 3 presents our findings using paired row averaging at several functional boundaries of the human methylome.

#### Model-based matrix completion

To enable the unbiased estimation of methylation tendencies across region types, while maintaining site-specific resolution, we developed COMPARE (COMparison of Phenotypes Averaged by REgion). COMPARE is a sparse-matrix completion technique that determines for each entry of an aggregation matrix a “methylation tendency” that reflects the extent to which the site corresponding to that entry would be methylated, if the DNA at the site could undergo methylation. COMPARE uses logistic-regression to perform inference of site-specific methylation tendencies and incorporates the regional observed methylation values and extent of missing data. The inferred methylation tendency is computed for each location (*i*,*j*) of an aggregation matrix as

$$ {M}_{i,j}=\frac{1}{1+{e}^{-\left({b}_j{B}_{i,j}+{x}_j{X}_{i,j}+{y}_j{Y}_{i,j}+{z}_j\right)}} $$

where *X*_*i*,*j*_ is the average methylation rate of row *i* excluding site (*i*,*j*), *Y*_*i*,*j*_ is the proportion of sites in row *i* with missing data (excluding site (*i*,*j*)), *B*_*i*,*j*_ is a binary variable for the presence of any methylatable sites in row *i* (excluding site (*i*,*j*)), and *b*_*j*_, *x*_*j*_,*y*_*j*_ and *z*_*j*_ are column-specific parameters learned from the non-missing matrix values (see Methods for further details). When analyzing large genomic segments or segments that are likely to span different conservation patterns *X*_*i*,*j*_, *Y*_*i*,*j*_ and *B*_*i*,*j*_ are determined from a user-defined restricted region around each relative location (e.g. 100 bp) rather than the entire region span (Additional file 1: Fig. S4). COMPARE’s inference procedure results in a complete matrix, allowing average-based statistics such as *C*, *R* and *M* to be used (Fig. 4). Importantly, COMPARE’s inference approach assigns a different set of parameters for every column, enabling characterization and comparison of DNA methylation tendencies at base pair resolution (Fig. 3, CpG island panel).

We tested COMPARE’s predictive ability by performing 10-fold cross validation on exon, intron, 5′-UTR and coding regions. COMPARE’s estimates were highly correlated with the bisulfite-measured methylation states at observed sites, in all four types of regions (Additional file 1: Fig. S5). As expected, the majority of sites in 5′UTR and coding regions are unmethylated, due to a high overlap of CG islands with such regions, while the majority of sites at mid-gene intron-exon junctions are methylated. In each run, COMPARE conducts a cross-validation analysis (using the non-missing matrix values) and reports the correlation between known and inferred values, assigning a precision measure to the specific dataset being analyzed.

#### Re-analysis of methylation tendencies across functional boundaries

Using COMPARE and paired-region averaging we could obtain an accurate view of the (average) extent of methylation across functional boundaries in the human methylome (Fig. 3). A reanalysis of 5′UTR-coding boundaries with COMPARE revealed that in contrast to previous reports, there is no significant difference in methylation tendency across the boundaries (*p* = 0.20), with a difference in methylation tendency of 0.01 (agreeing with the paired-row analysis that shows a difference of 0.02 and *p*-value of 0.09). An uncorrected %mCG analysis of the same data shows a false-positive seemingly significant (*p* = 4e-08) difference of 0.05 (Fig. 3). Additionally, methylation tendencies at both sides of the boundary were inferred by COMPARE to be higher than those measured by %mCG (Fig. 3). It is expected that the %mCG measures are lower than the true methylation tendencies because of the lower representation of CG sites at methylated regions.

An analysis of the intron-exon junctions of the human methylome with both COMPARE and paired-region averaging revealed a difference in methylation tendency of 0.05 (Fig. 3), approximately half of the difference observed with no correction (0.09). Interestingly, in Arabidopsis we observe an average difference of 0.03 at intron-exon junctions when no correction is applied to the whole-genome bisulfite data generated in [47], and a correction with COMPARE reveals an average difference of 0.04 (Additional file 1: Fig. S6) (in Arabidopsis the YS effect is not as pronounced as in human because the majority of the genome is unmethylated). We conclude that differences in methylation tendencies are similar in human and Arabidopsis at intron-exon junctions.

## Discussion

We have discovered a problem with the standard approach to analyzing DNA methylation at functional regions from genome-wide data and have shown that it affects comparative studies, resulting in false and exaggerated annotation of methylation differences in the human methylome. We established that averaging over CGs (or more generally sites that can undergo methylation) to account for data sparsity creates a bias due to the different distributions of the frequency of sites that can undergo methylation in both methylated and unmethylated regions. We have examined three averaging statistics that have been used previously and have shown that they can be arbitrarily different from one another, and that significant differences between them are observed in the analysis of the human methylome.

**Fig. 5:**
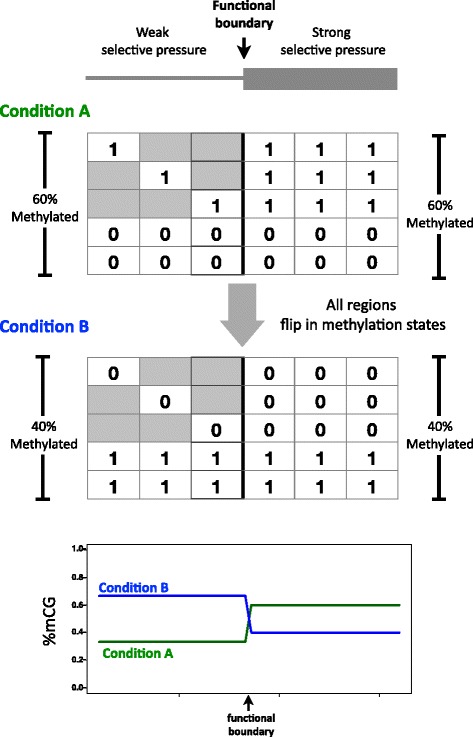
The YS effect can bias comparative studies of DNA methylation across different conditions and cell types. The matrices above represent a hypothetical setting of a functional boundary and the methylation rates measured at the different genomic locations, as specified in Fig. 1 (each row corresponds to a hypothetical instance, e.g. a specific intron-exon junction, and each column to a relative location in relation to the boundary analyzed, e.g. +1 from the intron-exon boundary). This example shows that, due to the YS effect, regional changes in DNA methylation intensity that are not associated with a functional boundary can be misleading when analyzed with the %mCG statistic

Most importantly, we have shown that comparative methylation studies are prone to biases due to a link between selection pressure and the extent of uneven distribution of CGs across regions due to different methylation rates. The link between the uneven distribution of data and the resulting bias is explained by the Yule-Simpson effect. Interestingly, even though the bias as we’ve characterized it is a special case of a known phenomenon, it occurs due to a different type of false intuition than is usually the case with Simpson’s paradox. Notably, the YS effect we report on can also arbitrarily bias comparative studies of the relative change in DNA methylation across different conditions (Fig. 5 and Additional file 2: Supplementary text, proposition 3). For example, it has been shown that in cancer cells the human genome becomes hypomethylated, but many CpG islands gain DNA methylation [48], a scenario similar to that illustrated in Fig. 5.

To account for this underlying bias and enable the sound comparison of regions of different types we suggest using one of two procedures. Paired-region averaging allows unbiased regional comparisons, while compromising site-specific resolution. An additional limitation of paired-region averaging is that it is strictly pairwise-comparative, and cannot asses the quantitative methylation tendency of a region type. The second method we propose makes use of a model-based matrix completion technique. Our developed method, COMPARE, is an inference procedure that uses structured logistic regression for sparse-matrix completion. We have shown that COMPARE achieves high accuracy for instances of the human methylome. Analyses with these two methods provide novel conclusions, such as that the difference in methylation at 5′UTR-Coding junctions is small and insignificant, and that the difference in methylation at intron-exon junctions is of similar magnitude in human and Arabidopsis. Additionally, COMPARE’s inference approach maintains site-specific inference, allowing the detection of gradually changing methylation rates, or local methylation tendency.

### Testing for the presence of the YS effect in other genomic settings

In this work we have focused on biases in methylation studies that are present in the human genome. Other organisms have been shown to have different genome-wide DNA methylation patterns than human [8, 13] and it will be interesting to test the extent to which the YS effect is present in comparison studies of methylomes across other organisms.

It will be interesting to characterize the extent of the YS effect we report on in analyses applied to 5-hydroxymethylcytosine (5hmC) data. We have shown here that the sparsity and uneven distribution of CG sites due to deamination biases averaging-based studies of 5hmC that have attempted to determine 5hmC rates in close proximity to exon-intron junctions. An annotation of the dependencies between 5hmC rates and CG site frequency and exploration of the applicability of COMPARE to such settings will be an interesting avenue for further investigation.

The YS effect is present in DNA methylation studies due to the process of deamination eliminating data in a manner that is associated with the extent of methylation. The association of the probability of observing data with functional type is present in other types of settings and can confound other genome-wide analyses. For example, in conservation analysis, gaps in alignment are frequently treated as missing data when computing conservation scores [49], but misalignments are less likely to occur at regions of high conservation [50] (Additional file 1: Fig. S9). Therefore, missing data is unevenly distributed across multiple sequence alignments, and ignoring it is likely to lead to biased conservation scores, in which the scores computed at a region with misalignments are an over-estimate of the true conservation tendency. In other settings the appearance of the YS effect may be more subtle and harder to characterize, but may bias comparative studies nonetheless.

To detect instances in which the Yule-Simpson effect is underlying observed differences across regions we propose conducting randomization tests in which the values of observed data-points are randomized but not the locations at which there is observed or missing data (see Methods). Observing an uneven distribution of the statistic studied across regions following such randomizations is an indication that the YS effect is present in the analyses conducted (Fig. 3, leftmost column). We have implemented such a randomization feature within the COMPARE package that can be used for arbitrary datasets. The generalization of COMPARE and Paired-region averaging to additional instances within the genomics field will be an interesting direction to explore.

## Conclusions

Seemingly straightforward averaging approaches may be heavily biased by an underlying non-uniformity in the distribution of the data. This phenomenon has been identified in the field of statistics as the Yule-Simpson effect, and we report here on its presence in genome-wide analyses, focusing on DNA methylation studies. We present two methods (a parametric and non-parametric approach) for bias correction and show that a corrected analysis eliminates previously reported differences in DNA methylation across functional boundaries. We conclude that genomic studies in which missing data is naïvely ignored during averaging should be carefully assessed for non-uniformity of the missing data, and we have presented a method for doing so. Additionally, the methods we have introduced for correction of the YS effect should be applicable not only to DNA methylation studies but to any genome-wide anlayses where averaging is performed.

## Methods

### Human DNA methylation data

We downloaded the publicly available bisulfite sequencing dataset for IMR90 cells from [14] and computed a methylation rate for each CG site overlapped by at least three reads. The rate was estimated according to the proportion of overlapping reads that were methylated. We used the Ref-Seq gene annotation for hg18 downloaded from the UCSC browser [51] for the determination of types of functional regions.

The intron-exon junctions analyzed here were defined to be intron-exon junctions at intragenic exons of the Ref-Seq gene annotation such that the exon is not the first or last exon of any transcript of the Ref-Seq annotation, the intron and exon are at least 200 bps long, the intronic region analyzed (200 bps from the junction) does not overlap a Ref-Seq annotated exon, and the exon region considered does not overlap with any Ref-Seq intron-exon junction.

The 5′UTR-Coding junctions analyzed were taken to be 5′UTR-coding junctions in the Ref-Seq annotation (hg18), such that the coding start site is determined as complete (cmpl), and the 100 bps around the junction are on an annotated exon.

The CpG island junctions analyzed were taken to be regions 300 bp upstream and 300 bp downstream from the upstream boundary of each CpG island (annotation hg18, downloaded from the UCSC Genome Browser website). Junctions considered were those for which the associated CpG island was longer than 300 bps, and the upstream preceding region did not overlap any annotated CpG island.

In Table 1 the regions analyzed were the intron and exon regions of the 200 bps intron-exon junctions described above, and the promoter regions were taken as 500 bps regions upstream from the Ref-Seq TSSs, which did not overlap with any gene regions from that annotation.

The enhancer regions in Table 1 were taken to be the 7457 regions determined by ChromHMM [52] as being in a “4 Strong Enhancer” state for Normal Human Lung Fibroblasts (NHLF) downloaded using the UCSC Table tool [51] (a ChromHMM track for IMR90 is currently unavailable), and which were at least 500 bp long. A region of length 500 bp, centered around the center of the enhancer, was taken from each enhancer region.

Genomic locations for the motif of the p53 transcription factor (TF) were downloaded from the Motifmap website (M00034) (http://motifmap.ics.uci.edu/) [53]. The motif region for p53 contains a CG site, is relatively long (21 bp), and 553 occurrences of the motif were detected across the human genome.

For the reanalysis of [23], datasets were downloaded from the GEO database, and the six chromosomes for which data was available were analyzed.

All statistical significance tests were conducted using the R package.

### Regional average correction for 5′UTR-coding junctions

For a fixed distance from the junction (100 bps) the mean methylation rate of each individual 5′UTR and its corresponding coding region was computed. Then, any coupled instances that did not both have an assigned value (due to the lack of CG sites) were discarded. This last step was taken in order to correct for the Yule-Simpson effect (see paired-region averaging correction method in text). The resulting 2977 × 2 matrix had no missing elements, and the methylation values were compared using the Mann–Whitney-Wilcoxon rank test.

### Simulating equal methylation tendency

To simulate datasets with equal methylation tendency across a functional boundary (Fig. 2 middle row and Fig. 3 left panel), we constructed a joint matrix by concatenating the two given matrices (one for each side of the junction) while maintaining the coupling of the rows. The new constructed matrix contained in each row a continuous genome fragment that crosses the boundary (as illustrated in Fig. 1). We then randomly permuted the values of the CG sites in each row of the union-matrix, keeping the structure of the rows–the sites that contain values and those that are empty–fixed. For example, a row that was originally (XX1XX||XX0X0) would be perturbed to either (XX0XX||XX1X0), (XX0XX||XX0X1), or (XX1XX||XX0X0) with equal probability.

### Simulated methylation states across a functional boundary

To generate a dataset with known differences in methylation and conservation rates across a functional boundary, analyzed in Fig. 2, we used the intragenic intron-exon junction set described previously as a basis, and generated a dataset in which each junction region was set to have all CG instances be 1 or 0, if the mean of the values at the row was larger or equal to 0.5 or smaller than 0.5, respectively. Then, 5 % of the methylated rows were randomly selected to be differentially methylated, and at those rows the intron-side CG instances were changed to 0. This resulted in a simulated dataset in which 3.2 % of the regions were differentially methylated. Finally, due to the very low frequency of values close to the boundary at intron-exon junctions, in this simulated dataset we substituted at each side the three columns closest to the boundaries with their adjacent three columns.

### COMPARE design and implementation

In order to correct for the YS effect and enable the assessment and comparison of DNA methylation tendencies at site-specific resolution we have developed COMPARE. Given an aggregation matrix, COMPARE infers at each location a methylation tendency: the probability that it would be methylated if it were to have a methylatable site (CG site).

COMPARE infers methylation tendencies using a logistic regression sparse-matrix completion approach. Given an aggregation matrix ***W***, COMPARE estimates a set of parameters for each of ***W***’s columns, and infers site-specific methylation tendencies. Additionally, COMPARE conducts a 10-fold cross-validation using the observed (non-missing) values of ***W*** and outputs accuracy estimates.

Formally, let ***W***_*i*,(−*j*)_ be row *i* of matrix ***W***, excluding the element at column *j* (*W*_*i*,*j*_). The following three features are associated with each location (*i*,*j*):

1. *B*_*i*,*j*_ - A binary variable indicating whether ***W***_*i*,(−*j*)_ has no methylation values.
2. *X*_*i*,*j*_ - The average methylation rate at ***W***_*i*,(−*j*)_, or 0 if *B*_*i*,*j*_ = 1.
3. *Y*_*i*,*j*_ - The proportion of sites with missing data in ***W***_*i*,(−*j*)_.

At each column *j*, parameters *b*_*j*_, *x*_*j*_,*y*_*j*_ and *z*_*j*_ are estimated for a logistic regression model, using as a training set all non-missing values in column *j* (together with their features). Having all features and estimated parameters in place, the posterior methylation tendency of each site (*i*,*j*) is inferred using the logistic function:

$$ {M}_{i,j}=\frac{1}{1+{e}^{-\left({b}_j{B}_{i,j}+{x}_j{X}_{i,j}+{y}_j{Y}_{i,j}+{z}_j\right)}} $$

COMPARE can also generate features using a block-size specified by the user, rather than the entire region (matrix row), enabling meta-region analyses (e.g., Additional file 1: Fig. S4). COMPARE requires a minimum of ten or more observations at a given relative location (matrix column) to make inferences for that location, is implemented in R and is easily parallelizable. COMPARE’s running time depends on the number of regions analyzed and the number of relative locations (the size of the input matrix); running time for the 5′UTR regions (4852) at 100 bp resolution is <1 min on a standard laptop.

### Testing for presence of the YS effect

To test if the distribution of missing data across an analyzed dataset results in the YS effect, the following randomization test is implemented in COMPARE: for a given matrix in which all data is incorporated (rows being genomic instances and values being measured values at the specific genomic locations), the values in each row are randomly permuted while the locations with missing values are kept in tact. For example, a row that is (XX1XX||XX0X0) could be perturbed to either (XX0XX||XX1X0), (XX0XX||XX0X1), or (XX1XX||XX0X0) with equal probability. Following this randomization, column-averages are computed and plotted. If column-averages across the analyzed regions are significantly different from each other in the randomized datasets (Fig. 2 middle row and Fig. 3 left panel), these differences are due to the YS effect (the unequal distribution of missing data).

COMPARE can be freely downloaded at: http://bio.math.berkeley.edu/COMPARE/.

### Cross-validation analysis

In each run of COMPARE a ten-fold cross-validation was conducted to assess the overall accuracy. The observed (non missing) variables of each column were randomly split into ten sets. In each iteration one of the ten sets was taken out of the training set, parameters were estimated, and methylation tendencies were inferred for the taken-out set. All inferences were then pooled together to obtain accuracy estimates.

After a cross-validation run, the observed estimates of the methylation tendencies–the proportion of methylated reads overlapping a location in the case of bisulfite–are compared to the inferred methylation tendencies (as shown in Fig. 4). Since both estimates contain errors (due to model inaccuracies and the relatively low coverage of the bisulfite data), our regression line minimizes the sum-of-squares of the distances from the points to their projection on the line using the first principal component of the two-dimensional data [54]. COMPARE reports the slope of the regression line and the extent of variance in the data explained by the regression as compared to a random line.

## Additional files

Additional file 1:
**Supplementary figures.**
Additional file 2:
**Supplementary text.**

## Footnotes

1 Our analysis generalizes to different types of sites (such as CHH and CHG, or simply C) but for the sake of simplicity in this manuscript we restrict the discussion to CGs.

## References

[1] Chinwalla AT, Cook LL, Delehaunty KD, Fewell GA, Fulton LA, Fulton RS, Graves TA, Hillier LW, Mardis ER, McPherson JD, Miner TL, Nash WE, Nelson JO, Nhan MN, Pepin KH, Pohl CS, Ponce TC, Schultz B, Thompson J, Trevaskis E, Waterston RH, Wendl MC, Wilson RK, Yang S-P, An P, Berry E, Birren B, Bloom T, Brown DG, Butler J (2002). Initial sequencing and comparative analysis of the mouse genome. Nature.

[2] Doolittle WF, Fraser P, Gerstein MB, Graveley BR, Henikoff S, Huttenhower C, Oshlack A (2013). Sixty years of genome biology. Genome Biol.

[3] Guo H, Zhu P, Yan L, Li R, Hu B, Lian Y, Yan J, Ren X, Lin S, Li J, Jin X, Shi X, Liu P, Wang X, Wang W, Wei Y, Li X, Guo F, Wu X, Fan X, Yong J, Wen L, Xie SX, Tang F, Qiao J (2014). The DNA methylation landscape of human early embryos. Nature.

[4] Su J, Wang Y, Xing X, Liu J, Zhang Y (2014). Genome-wide analysis of DNA methylation in bovine placentas. BMC Genomics.

[5] Liang D, Zhang Z, Wu H, Huang C, Shuai P, Ye C-Y, Tang S, Wang Y, Yang L, Wang J, Yin W, Xia X (2014). Single-base-resolution methylomes of populus trichocarpa reveal the association between DNA methylation and drought stress. BMC Genet.

[6] Zhong S, Fei Z, Chen Y-R, Zheng Y, Huang M, Vrebalov J, McQuinn R, Gapper N, Liu B, Xiang J, Shao Y, Giovannoni JJ (2013). Single-base resolution methylomes of tomato fruit development reveal epigenome modifications associated with ripening. Nat Biotechnol.

[7] Gao F, Liu X, Wu X-P, Wang X-L, Gong D, Lu H, Xia Y, Song Y, Wang J, Du J, Liu S, Han X, Tang Y, Yang H, Jin Q, Zhang X, Liu M (2012). Differential DNA methylation in discrete developmental stages of the parasitic nematode Trichinella spiralis. Genome Biol.

[8] Zemach A, McDaniel IE, Silva P, Zilberman D (2010). Genome-wide evolutionary analysis of eukaryotic DNA methylation. Science.

[9] Sati S, Tanwar VS, Kumar KA, Patowary A, Jain V, Ghosh S, Ahmad S, Singh M, Reddy SU, Chandak GR, Raghunath M, Sivasubbu S, Chakraborty K, Scaria V, Sengupta S (2012). High resolution methylome map of rat indicates role of intragenic DNA methylation in identification of coding region. PLoS One.

[10] Gent JI, Ellis NA, Guo L, Harkess AE, Yao Y, Zhang X, Dawe RK (2013). CHH islands: de novo DNA methylation in near-gene chromatin regulation in maize. Genome Res.

[11] Hsieh T-F, Ibarra CA, Silva P, Zemach A, Eshed-Williams L, Fischer RL, Zilberman D (2009). Genome-wide demethylation of arabidopsis endosperm. Science.

[12] Lister R, O’Malley RC, Tonti-Filippini J, Gregory BD, Berry CC, Millar AH, Ecker JR (2008). Highly integrated single-base resolution maps of the epigenome in arabidopsis. Cell.

[13] Feng S, Cokus SJ, Zhang X, Chen P-Y, Bostick M, Goll MG, Hetzel J, Jain J, Strauss SH, Halpern ME, Ukomadu C, Sadler KC, Pradhan S, Pellegrini M, Jacobsen SE (2010). Conservation and divergence of methylation patterning in plants and animals. Proc Natl Acad Sci.

[14] Lister R, Pelizzola M, Dowen RH, Hawkins RD, Hon G, Tonti-Filippini J, Nery JR, Lee L, Ye Z, Ngo Q-M, Edsall L, Antosiewicz-Bourget J, Stewart R, Ruotti V, Millar AH, Thomson JA, Ren B, Ecker JR (2009). Human DNA methylomes at base resolution show widespread epigenomic differences. Nature.

[15] Wang X, Wheeler D, Avery A, Rago A, Choi J-H, Colbourne JK, Clark AG, Werren JH (2013). Function and evolution of DNA methylation in nasonia vitripennis. PLoS Genet.

[16] Zilberman D, Gehring M, Tran RK, Ballinger T, Henikoff S (2007). Genome-wide analysis of Arabidopsis thaliana DNA methylation uncovers an interdependence between methylation and transcription. Nat Genet.

[17] Ball MP, Li JB, Gao Y, Lee J-H, LeProust EM, Park I-H, Xie B, Daley GQ, Church GM (2009). Targeted and genome-scale strategies reveal gene-body methylation signatures in human cells. Nat Biotechnol.

[18] Zhang X, Yazaki J, Sundaresan A, Cokus S, Chan SW-L, Chen H, Henderson IR, Shinn P, Pellegrini M, Jacobsen SE, Ecker JR (2006). Genome-wide high-resolution mapping and functional analysis of DNA methylation in arabidopsis. Cell.

[19] Laurent L, Wong E, Li G, Huynh T, Tsirigos A, Ong CT, Low HM, Sung KWK, Rigoutsos I, Loring J, Wei C-L (2010). Dynamic changes in the human methylome during differentiation. Genome Res.

[20] Hackett JA, Sengupta R, Zylicz JJ, Murakami K, Lee C, Down TA, Surani MA (2013). Germline DNA demethylation dynamics and imprint erasure through 5-hydroxymethylcytosine. Science.

[21] Flores KB, Amdam GV (2011). Deciphering a methylome: what can we read into patterns of DNA methylation?. J Exp Biol.

[22] Chen P-Y, Feng S, Joo JW, Jacobsen SE, Pellegrini M (2011). A comparative analysis of DNA methylation across human embryonic stem cell lines. Genome Biol.

[23] Khare T, Pai S, Koncevicius K, Pal M, Kriukiene E, Liutkeviciute Z, Irimia M, Jia P, Ptak C, Xia M, Tice R, Tochigi M, Moréra S, Nazarians A, Belsham D, Wong AHC, Blencowe BJ, Wang SC, Kapranov P, Kustra R, Labrie V, Klimasauskas S, Petronis A (2012). 5-hmC in the brain is abundant in synaptic genes and shows differences at the exon-intron boundary. Nat Struct Mol Biol.

[24] Gelfman S, Cohen N, Yearim A, Ast G (2013). DNA-methylation effect on co-transcriptional splicing is dependent on GC-architecture of the exon-intron structure. Genome Res.

[25] Huang Y, Chavez L, Chang X, Wang X, Pastor WA, Kang J, Zepeda-Martínez JA, Pape UJ, Jacobsen SE, Peters B, Rao A (2014). Distinct roles of the methylcytosine oxidases Tet1 and Tet2 in mouse embryonic stem cells. Proc Natl Acad Sci.

[26] Choi JK (2010). Contrasting chromatin organization of CpG islands and exons in the human genome. Genome Biol.

[27] Flores K, Wolschin F, Corneveaux JJ, Allen AN, Huentelman MJ, Amdam GV (2012). Genome-wide association between DNA methylation and alternative splicing in an invertebrate. BMC Genomics.

[28] Choi JK, Bae J-B, Lyu J, Kim T-Y, Kim Y-J (2009). Nucleosome deposition and DNA methylation at coding region boundaries. Genome Biol.

[29] Regulski M, Lu Z, Kendall J, Donoghue MTA, Reinders J, Llaca V, Deschamps S, Smith A, Levy D, McCombie WR, Tingey S, Rafalski A, Hicks J, Ware D, Martienssen RA (2013). The maize methylome influences mRNA splice sites and reveals widespread paramutation-like switches guided by small RNA. Genome Res.

[30] Deaton AM, Bird A (2011). CpG islands and the regulation of transcription. Genes Dev.

[31] Blyth CR (1972). On Simpson’s paradox and the sure-thing principle. J Am Stat Assoc.

[32] Good IJ, Mittal Y (1987). The amalgamation and geometry of two-by-two contingency tables. Ann Stat.

[33] Simpson EH. The interpretation of interaction in contingency tables. J R Stat Soc Ser B Methodol. 1951;238–241.

[34] Yule GU (1903). Notes on the Theory of Association of Attributes in Statistics. Biometrika.

[35] Pearl J (1999). Simpson’s paradox: an anatomy. Causality: models, reasoning and inference.

[36] Bickel PJ, Hammel EA, O’Connell JW (1975). Sex bias in graduate admissions: data from berkeley. Science.

[37] Chi EC, Zhou H, Chen GK, Vecchyo DOD, Lange K (2013). Genotype imputation via matrix completion. Genome Res.

[38] Zhang M, Xie S, Dong X, Zhao X, Zeng B, Chen J, Li H, Yang W, Zhao H, Wang G, Chen Z, Sun S, Hauck A, Jin W, Lai J (2014). Genome-wide high resolution parental-specific DNA and histone methylation maps uncover patterns of imprinting regulation in maize. Genome Res.

[39] Bell JT, Pai AA, Pickrell JK, Gaffney DJ, Pique-Regi R, Degner JF, Gilad Y, Pritchard JK (2011). DNA methylation patterns associate with genetic and gene expression variation in HapMap cell lines. Genome Biol.

[40] Chodavarapu RK, Feng S, Bernatavichute YV, Chen P-Y, Stroud H, Yu Y, Hetzel JA, Kuo F, Kim J, Cokus SJ, Casero D, Bernal M, Huijser P, Clark AT, Krämer U, Merchant SS, Zhang X, Jacobsen SE, Pellegrini M (2010). Relationship between nucleosome positioning and DNA methylation. Nature.

[41] Feldmann A, Ivanek R, Murr R, Gaidatzis D, Burger L, Schübeler D (2013). Transcription factor occupancy can mediate active turnover of DNA methylation at regulatory regions. PLoS Genet.

[42] Kobayashi H, Sakurai T, Miura F, Imai M, Mochiduki K, Yanagisawa E (2013). High-resolution DNA methylome analysis of primordial germ cells identifies gender-specific reprogramming in mice. Genome Res.

[43] Molaro A, Hodges E, Fang F, Song Q, McCombie WR, Hannon GJ, Smith AD (2011). Sperm methylation profiles reveal features of epigenetic inheritance and evolution in primates. Cell.

[44] Akalin A, Garrett-Bakelman FE, Kormaksson M, Busuttil J, Zhang L, Khrebtukova I, Milne TA, Huang Y, Biswas D, Hess JL, Allis CD, Roeder RG, Valk PJM, Löwenberg B, Delwel R, Fernandez HF, Paietta E, Tallman MS, Schroth GP, Mason CE, Melnick A, Figueroa ME (2012). Base-pair resolution DNA methylation sequencing reveals profoundly divergent epigenetic landscapes in acute myeloid leukemia. PLoS Genet.

[45] Cokus SJ, Feng S, Zhang X, Chen Z, Merriman B, Haudenschild CD, Pradhan S, Nelson SF, Pellegrini M, Jacobsen SE (2008). Shotgun bisulphite sequencing of the Arabidopsis genome reveals DNA methylation patterning. Nature.

[46] Wang X, Duan C-G, Tang K, Wang B, Zhang H, Lei M, Lu K, Mangrauthia SK, Wang P, Zhu G, Zhao Y, Zhu J-K (2013). RNA-binding protein regulates plant DNA methylation by controlling mRNA processing at the intronic heterochromatin-containing gene IBM1. Proc Natl Acad Sci.

[47] Zemach A, Kim MY, Hsieh P-H, Coleman-Derr D, Eshed-Williams L, Thao K, Harmer SL, Zilberman D (2013). The Arabidopsis nucleosome remodeler DDM1 allows DNA methyltransferases to access H1-containing heterochromatin. Cell.

[48] Ehrlich M (2002). DNA methylation in cancer: too much, but also too little. Oncogene.

[49] Simmons MP, Ochoterena H (2000). Gaps as characters in sequence-based phylogenetic analyses. Syst Biol.

[50] Tian D, Wang Q, Zhang P, Araki H, Yang S, Kreitman M, Nagylaki T, Hudson R, Bergelson J, Chen J-Q (2008). Single-nucleotide mutation rate increases close to insertions/deletions in eukaryotes. Nature.

[51] Karolchik D, Hinrichs AS, Furey TS, Roskin KM, Sugnet CW, Haussler D, Kent WJ (2004). The UCSC Table Browser data retrieval tool. Nucleic Acids Res.

[52] Ernst J, Kellis M (2012). ChromHMM: automating chromatin-state discovery and characterization. Nat Methods.

[53] Daily K, Patel VR, Rigor P, Xie X, Baldi P (2011). MotifMap: integrative genome-wide maps of regulatory motif sites for model species. BMC Bioinformatics.

[54] Bishop CM (2006). Pattern recognition and machine learning. Volume 1.

