# Supplementary material for: Controlling for conservation in genome-wide DNA methylation studies
Source: BMC Genomics. 2015 May 30;16(1):420. doi: 10.1186/s12864-015-1604-3 (PMC4448855; doi:10.1186/s12864-015-1604-3)
Supplement: Additional file 1: — Supplementary figures. [file 12864_2015_1604_MOESM1_ESM.pdf]

(a)

	Female	Male
Applicants	550	550
Admitted	**28.2%**	**41.8%**

	Female		Male	
	Applicants	Admitted	Applicants	Admitted
Department A	150	**50%**	400	**50%**
Department B	400	**20%**	150	**20%**

(b)

	Females								Males							
	F1	F2	F3	F4	F5	F6	F7	F8	M1	M2	M3	M4	M5	M6	M7	M8
Department 1	0		0		0		0		0		0		0		0	
Department 2		0		0		0		0		0		0		0		0
Department 3	0		0		0		0		0		0		0		0	
Department 4		0		0		0		0		0		0		0		0
Department 5	1								1		1		1		1	
Department 6								1		1		1		1		1
Department 7						1			1		1		1		1	
Department 8			1							1		1		1		1
Department 9					1				1		1		1		1	
Department 10		1								1		1		1		1
Department 11							1		1		1		1		1	
Department 12				1						1		1		1		1

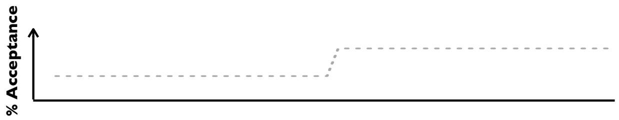

Figure S1: (a) A toy example for the YS effect in university admission rates as described in [27]. When tested for general admission rates (upper table) it was observed that the acceptance percentage for female applicants was considerably lower than that for male applicants. However when breaking down the statistics by departments (lower table) it was revealed that departments do not display gender-bias in acceptance rates. The lower total admission rate of female applicants is due to a difference in the application tendency between the sexes: females, in contrast to males, have an inclination to apply to departments with exceptionally low admissions rates. (b) A formalization of the example in Figure 1 as an instance of the Yule-Simpson effect. A value of 1 or 0 indicates applicant was accepted or rejected, respectively. If considering only the *C* measure for the two matrices, it would be wrong to conclude that the acceptance mechanism is biased against females from the observation that the female acceptance rates are significantly lower than those of males (shown at the bottom).

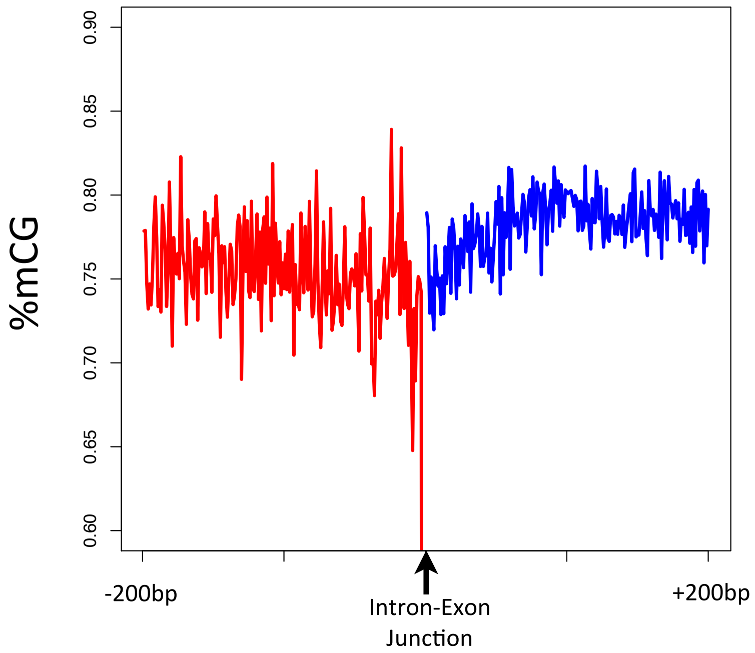

Figure S2: mCG/CG values for a subset of intron-exon junctions (n=16,407), for which either the intronic region's methylation average was larger than that of the exon by 0.1, or one of the regions has no methylation values. The difference observed in the mCG/CG rates of this plot is not in concordance with the average methylation values of the rows, which reveals an opposite trend in which the intronic regions are methylated to a higher extent than the exonic regions (C_{Intron} =0.75, R_{Intron}=0.82 ; C_{Exon} =0.78, R_{Exon}=0.78).

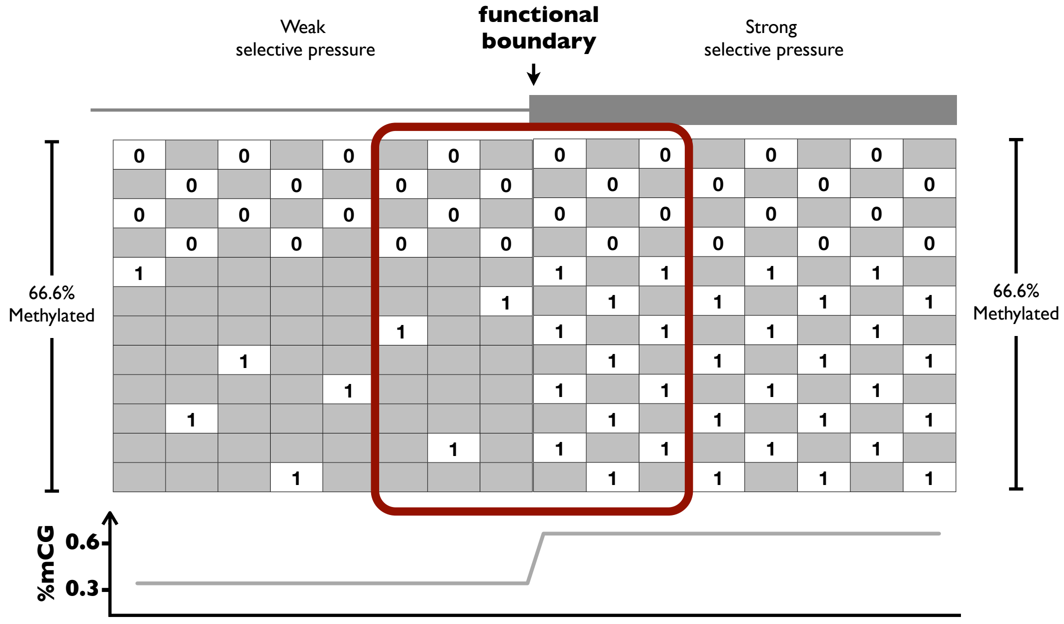

Figure S3: A toy-example illustrating the need to discard junction instances at which a row-average cannot be computed for either or both sides of the junction. When considering the region marked in red to determine whether methylation tendency changes across the functional boundary, the paired-region averaging method would result in identical distribution across the boundary (3 rows would have an average of 1 and 3 rows an average of 0, the R value for both sides would be 0.5). However, if one does not discard the regions at which the left side had no values, the distributions of the row averages would differ.

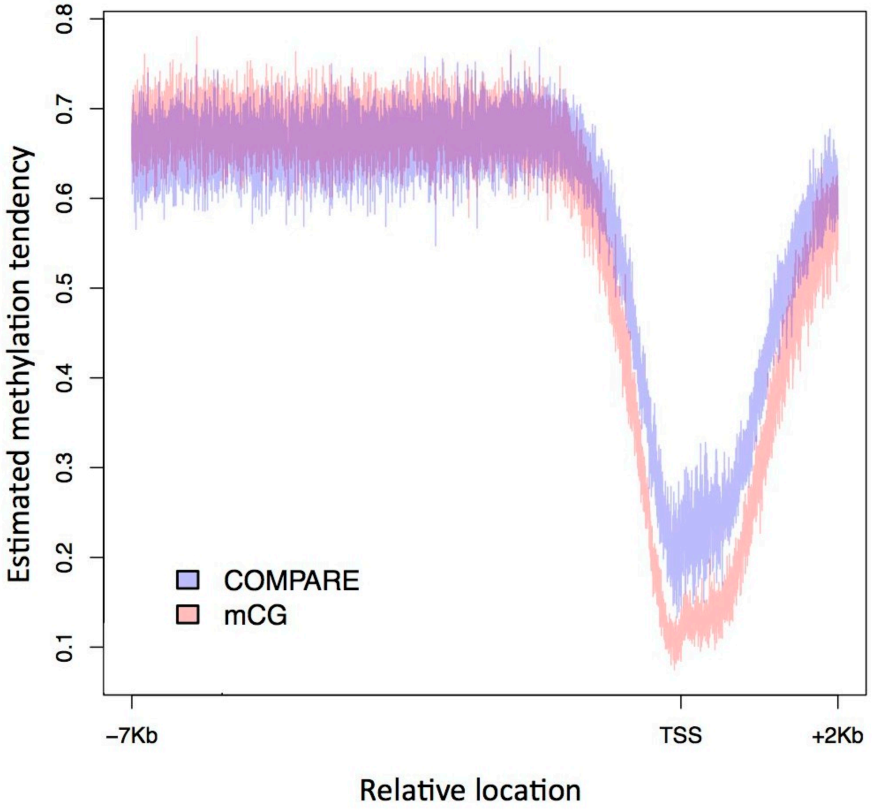

Figure S4: A meta-analysis of DNA methylation extents around promoter regions as measured using the mCG/CG measure (red) and the corrected COMPARE analysis annotating DNA methylation tendencies (blue). The corrected analysis shows a shallower dip around the TSS sites than that of the uncorrected mCG analysis, and a "step-wise" behavior at the TSS site seen when using the mCG measure vanishes in the corrected analysis and is therefore probably due to the change in conservation rates rather than in DNA methylation tendencies. This analysis used COMPARE's meta-analysis module assigning features to each relative location based on its 200bp surrounding.

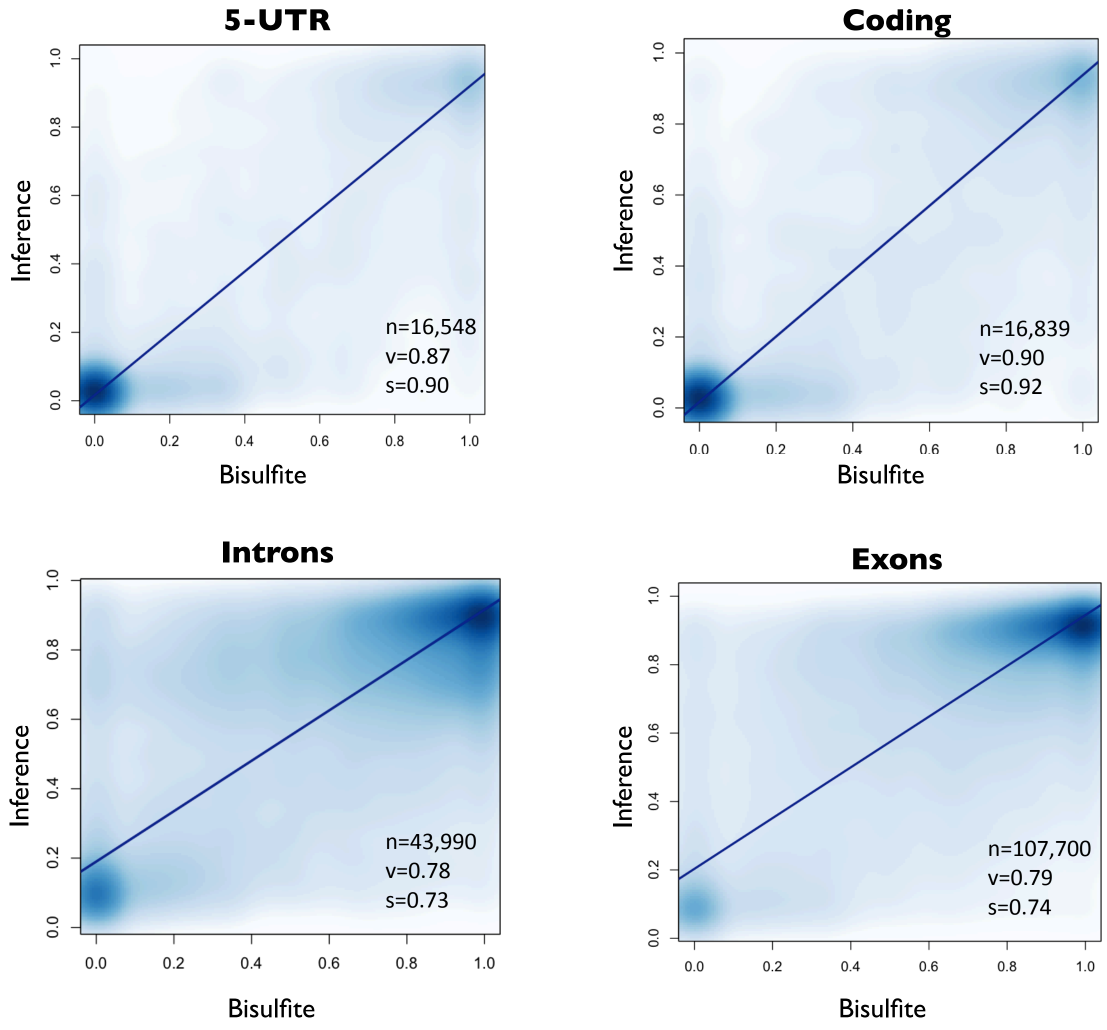

Figure S5: Analysis with COMPARE corrects for the Yule-Simpson effect. Shown are results of ten-fold cross-validations for 5'UTR, coding, intronic and exonic regions, at the corresponding junctions analyzed in Figure 3. Smoothing was used to display the large number of data points. In each plot *n* is the number of data points in the matrix and the regression line is shown in dark blue where *s* is its slope, and *v* is the additional amount of variance in the data explained by the regression line relative to a random line (Methods).

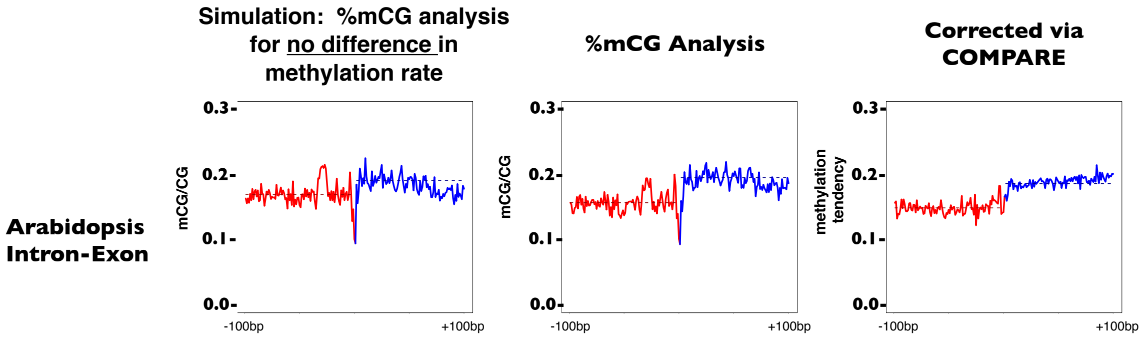

Figure S6: In the same format as in Figure 3, methylation values for intra-genic intron-exon junctions of Arabidopsis are shown. Dashed lines mark the means of each of the sides compared.

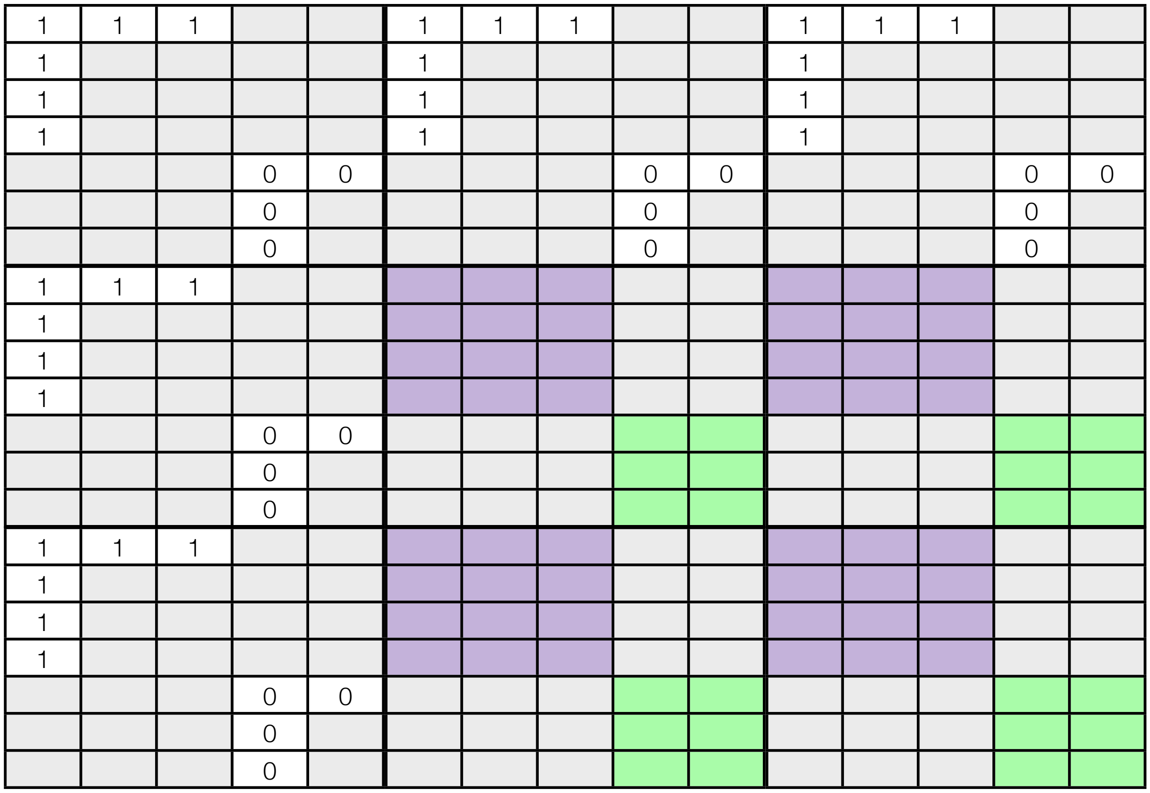

Figure S7: First a matrix **A** is constructed such that R_A=r and C_A=c (top left corner 7x5 matrix). Matrix **B** is constructed by concatenating k+1 matrices of type **A** for the upper rows and leftmost columns as shown. The rest of the matrix is filled with “missing value” instances. The locations colored purple are those that can be set to 1 and those colored green can be set to 0 without altering R_B and C_B.

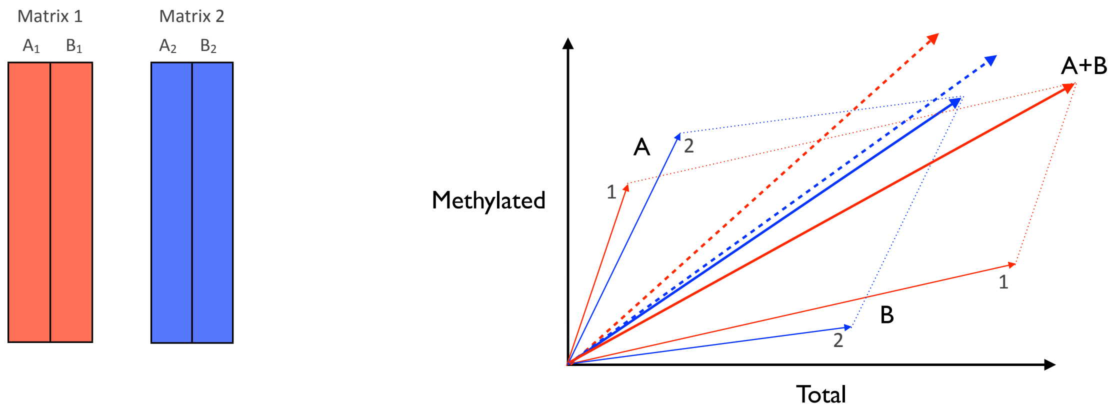

Figure S8: Geometric interpretation of Simpson's paradox. The x-axis represents the total number of elements considered (equivalent to the number of CG sites), and the y-axis corresponds to the numerator value (equivalent to the number of methylated CG sites). Using this representation, the slopes of the vectors for columns A_1, A_2, B_1 and B_2 are equal to the column-specific means. The slopes of the red and blue dashed lines are the averages of the slopes of the pair A_1 and B_1, and the pair A_2 and B_2, respectively, and correspond to measure C as defined in the manuscript. The slopes of the red and blue full lines marked as A+B correspond to the means over all (non-missing) values of matrix 1 and matrix 2, respectively, and correspond to measure M as defined in the manuscript. As can be seen, in this case the average of slopes is the reverse of the slope of averages.

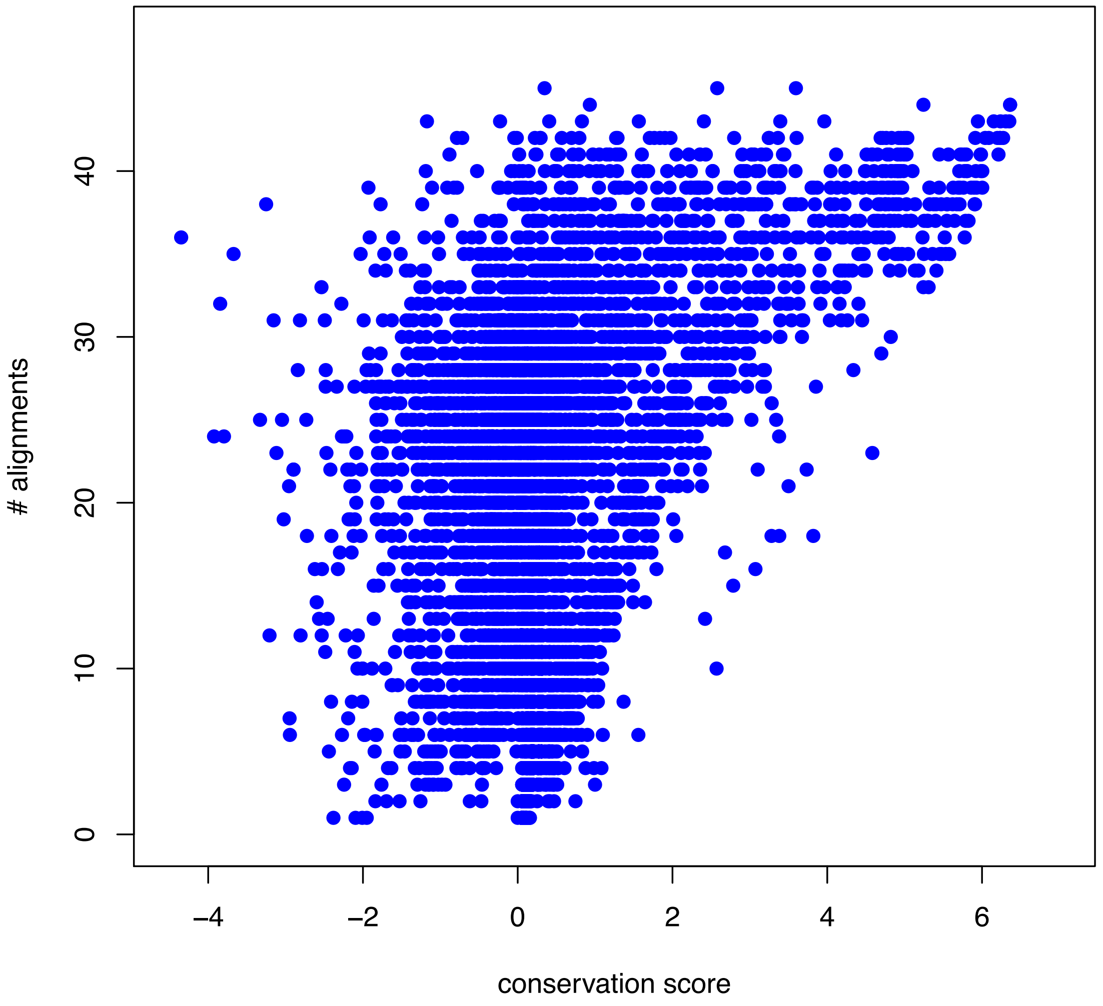

Figure S9: Conservation scores (PhyloP) and the number of successful alignments from the 45-vertebrate multiple-sequence alignment to the human genome are positively correlated (r=0.42). Conservation scores and number of successful alignments were determined for locations randomly chosen from coding, intronic and intergenic regions (2,000 locations chosen for each) from human chromosome 12.
