# Supplementary material for: Controlling for conservation in genome-wide DNA methylation studies
Source: BMC Genomics. 2015 May 30;16(1):420. doi: 10.1186/s12864-015-1604-3 (PMC4448855; doi:10.1186/s12864-015-1604-3)
Supplement: Additional file 2: — Supplementary text. [file 12864_2015_1604_MOESM2_ESM.pdf]

Supplementary information for “Variability of conservation rates casts doubt on DNA methylation differences at functional boundaries”

Meromit Singer and Lior Pachter

Supplementary text for Proposition 1

Proposition 1: For any three rational numbers $0 < r, c, m < 1$, there exists an aggregation matrix W such that $R_W = r$, $C_W = c$ and $M_W = m$.

Proof: The three given rational numbers can be written as $r = \frac{r_1}{(r_0+r_1)}$, $c = \frac{c_1}{(c_0+c_1)}$ and $m = \frac{m_1}{(m_0+m_1)}$, where r_0, r_1, c_0, c_1, m_0 and $m_1 \in \mathbb{N}$.

1. We first construct a matrix A for which $R_A = r$ and $C_A = c$. We construct an $(r_0 + r_1) \times (c_0 + c_1)$ matrix with all entries missing except for: $A_{1,1:c_1} = 1$, $A_{1:r_1,1} = 1$, $A_{(r_1+1):(r_1+r_0),(c_1+1)} = 0$ and $A_{(r_1+1),(c_1+1):(c_1+c_0)} = 0$ (top left corner matrix in Supplementary Fig. 7).

2. Next, we construct a matrix B using A such that $R_B = r$, $C_B = c$ and $M_B = m$. For any $k \in \mathbb{N}$, we can construct a $((k+1) \cdot (r_0+r_1)) \times ((k+1) \cdot (c_0+c_1))$ matrix B using A as a template by concatenating $k+1$ instances of A one alongside the other, "stacking" $k+1$ instances of A one on top of the other, and filling the rest of the matrix with missing values (as in Supplementary Fig. 7). Notice that by doing so $R_B = r$, $C_B = c$ and there are at least $k^2 r_0 c_0$ and $k^2 r_1 c_1$ values that can be set in B to be 0 (green, Supplementary Fig. 7) or 1 (purple, Supplementary Fig. 7), without changing R_B and C_B. In order to get $M_B = m$, we add 0s and 1s to B such that for some integer multiplier v, the number of 0s is $v \cdot m_0$ and the number of 1s is $v \cdot m_1$. Variables k and v that fulfill the following two criteria suffice:

$$(2k+1)(c_0 + r_0 - 1) \leq v \cdot m_0 \leq k^2 r_0 c_0.$$

and

$$(2k+1)(c_1 + r_1 - 1) \leq v \cdot m_1 \leq k^2 r_1 c_1.$$

We can find such k and v variables by finding k and v such that

$$3k(c_0 + r_0) \leq v \cdot m_0 \leq k^2 r_0 c_0,$$

and

$$3k(c_1 + r_1) \leq v \cdot m_1 \leq k^2 r_1 c_1$$

which is equivalent to requiring

$$k \cdot \max\left\{\frac{3(c_0 + r_0)}{m_0}, \frac{3(c_1 + r_1)}{m_1}\right\} \leq v \leq k^2 \cdot \min\left\{\frac{r_0 c_0}{m_0}, \frac{r_1 c_1}{m_1}\right\}.$$

This is always possible by choosing k to be sufficiently large.

Supplementary text for Proposition 2

Proposition 2: Let W_1 and W_2 be two aggregation matrices. $R_{W1} - R_{W2}$ can have opposite sign to $C_{W1} - C_{W2}$, and if it does then an instance of Simpson's paradox has occurred.

Proof: For any two aggregation matrices W_1 and W_2, Simpson's paradox occurs if either (or both) of the following occur:

1. $M_{W1} - M_{W2}$ has a different sign than $R_{W1} - R_{W2}$.

2. $M_{W1} - M_{W2}$ has a different sign than $C_{W1} - C_{W2}$.

In both of these cases, the underlying Yule-Simpson effect results in Simpson's paradox: the comparison of the aggregation over the rows (columns) results in an opposite trend than that observed when comparing the different rows (columns). If $R_{W1} - R_{W2}$ has the opposite sign of $C_{W1} - C_{W2}$, then either (1) or (2) must hold and therefore an instance of Simpson's paradox occurs (an example is seen in Supplementary Fig. 8).

Supplementary text for Proposition 3

Proposition 3: The difference in methylation rates between functional regions measured by column-averaging can change arbitrarily across experimental conditions, even if the difference in regional methylation rates remains unchanged. Formally, For any two pairs of rational numbers $0 < c_1, c_2 < 1$ and $0 < \hat{c}_1, \hat{c}_2 < 1$, there exists an aggregation matrix pair $< W_1, W_2 >$ and an M-transformation to $< \widehat{W_1}\ , \widehat{W_2} >$ such that :

1. $C_{W_1} = c_1$ and $C_{W_2} = c_2$
2. $C_{\widehat{W_1}} = \hat{c}_1$ and $C_{\widehat{W_2}} = \hat{c}_2$.

We will make use of the following three definitions in the proof:

Definition: Define an *aggregation matrix pair* $< W_1, W_2 >$ to be the by-row concatenation of two $n \times m$ aggregation matrices $< W_1, W_2 >$, such that each concatenated row displays at most one character type (aside from the missing character). (See sketch below)

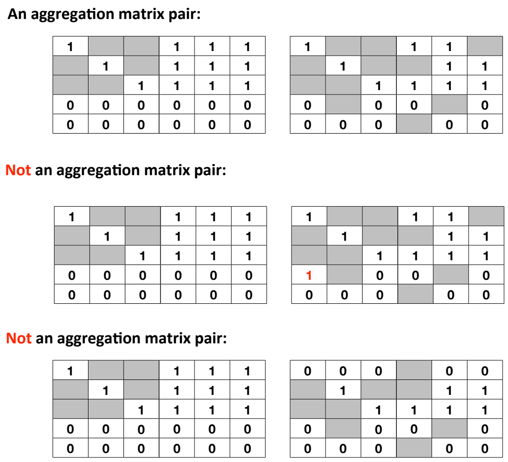

Definition: Let a *methylation-transformation* (M-transformation) of an aggregation matrix pair $< W_1, W_2 >$ be the reassignment of (non-missing) values in a subset of the rows of W_1 and W_2, to result in $\widehat{W_1}$ and $\widehat{W_2}$ such that $< \widehat{W_1}, \widehat{W_2} >$ is also an aggregation matrix pair. (see sketch below for an example of an M-transformation.)

An aggregation matrix pair <W1,W2>:

1			1	1	1
	1		1	1	1
		1	1	1	1
0	0	0	0	0	0
0	0	0	0	0	0

1			1	1	
	1			1	1
		1	1	1	1
0		0	0		0
0	0	0		0	0

An M-transformation of <W1,W2>:

0			0	0	0
	1		1	1	1
		1	1	1	1
0	0	0	0	0	0
0	0	0	0	0	0

0			0	0	
	1			1	1
		1	1	1	1
0		0	0		0
0	0	0		0	0

Operation $OP()$ (Definition): Define operation $OP(< W_1, W_2 >, W_{i \in \{1,2\}}, v, k)$, on the following input

1. an aggregation matrix pair $< W_1, W_2 >$,
2. a matrix, $W_{i \in \{1,2\}}$, one of the matrices of the aggregation matrix pair in (1),
3. a rational number v,
4. and an integer k.

Operation OP edits the two matrices of the aggregation matrix pair as follows (assume without loss of generality that $W_i = W_1$):

1. Append k rows of type $(v, v, \ldots, v)$ (complete row, no missing values) to matrix W_1.
2. Append k rows to W_2 such that each added row has at least one instance of v and each column in the block added has exactly one instance of v (the rest are missing values). Add columns to W_2 as needed, maintaining complete rows and 1-instance-per-column-in-block format (see sketch below, starting with two empty matrices).

It is easy to see that completing this operation is always possible.

$OP(< W_1, W_2 >, W_1, 0, 3)$

0	0	0	0		
0	0	0		0	
0	0	0			0

$OP(< W_1, W_2 >, W_2, 1, 4)$

0	0	0	0	0			
0	0	0	0		0		0
0	0	0	0			0	
1				1	1	1	1
	1			1	1	1	1
		1		1	1	1	1
			1	1	1	1	1

$OP(< W_1, W_2 >, W_2, 0, 2)$

0	0	0	0	0			
0	0	0	0		0		0
0	0	0	0			0	
1				1	1	1	1
	1			1	1	1	1
		1		1	1	1	1
			1	1	1	1	1
0		0		0	0	0	0
	0		0	0	0	0	0

Proof: Representing by a or $\hat{a}$ the number of 1s and by b or $\hat{b}$ the number of 0s, we can write $c_1 = \frac{a_1}{a_1+b_1}$ and $\hat{c_1} = \frac{\hat{a}_1}{\hat{a}_1+\hat{b}_1}$, such that $a_1 + b_1 = \hat{a}_1 + \hat{b}_1$, and $c_2 = \frac{a_2}{a_2+b_2}$ and $\hat{c_2} = \frac{\hat{a}_2}{\hat{a}_2+\hat{b}_2}$, such that $a_2 + b_2 = \hat{a}_2 + \hat{b}_2$.

We will construct an aggregation-matrix-pair and an M-transformation.
We distinguish between the following cases:

1. If (without loss of generality) $c_1 > \hat{c}_1$ and $c_2 < \hat{c}_2$, preform the following operations on $< W_1, W_2 >$, (starting with $W_1 = \phi$ and $W_2 = \phi$):

   (a) $OP(< W_1, W_2 >, W_1, 1, (a_1 - \hat{a}_1) + 1)$
   (b) $OP(< W_1, W_2 >, W_1, 1, \hat{a}_1 - 2)$
   (c) $OP(< W_1, W_2 >, W_1, 0, b_1 - 2)$
   (d) $OP(< W_1, W_2 >, W_2, 1, a_2 - 2)$
   (e) $OP(< W_1, W_2 >, W_2, 0, b_1 - (\hat{a}_2 - a_2) - 2)$
   (f) $OP(< W_1, W_2 >, W_2, 0, \hat{a}_2 - a_2 + 1)$

   In the final $< W_1, W_2 >$ pair, all columns of W_1 have mean c_1 and all columns of W_2 have mean c_2. Applying the following $M - transition$: switch all rows in "block" (a) from 1 to 0, and all rows in "block" (f) from 0 to 1, results in $< \widehat{W_1}\ , \widehat{W_2} >$, such that the column averages of $\widehat{W_1}$ and $\widehat{W_2}$ are $\hat{c}_1$ and $\hat{c}_2$, respectively.

2. If $c_1 > \hat{c}_1$ and $c_2 > \hat{c}_2$ preform the following operations on $< W_1, W_2 >$, (starting with $W_1 = \phi$ and $W_2 = \phi$):

   (a) $OP(< W_1, W_2 >, W_1, 1, (a_1 - \hat{a}_1) - 1)$
   (b) $OP(< W_1, W_2 >, W_1, 1, \hat{a}_1 - 1)$
   (c) $OP(< W_1, W_2 >, W_1, 0, b_1 - 1)$
   (d) $OP(< W_1, W_2 >, W_1, 1, (a_2 - \hat{a}_2) - 1)$
   (e) $OP(< W_1, W_2 >, W_1, 1, \hat{a}_2 - 1)$
   (f) $OP(< W_1, W_2 >, W_1, 0, b_2 - 1)$

   Applying the $M - transition$: switch all rows in "block" (a) from 1 to 0, and all rows in "block" (c) from 1 to 0, results in $< \widehat{W_1}\ , \widehat{W_2} >$, such that the column averages of $\widehat{W_1}$ and $\widehat{W_2}$ are $\hat{c}_1$ and $\hat{c}_2$, respectively.

3. If $c_1 < \hat{c}_1$ and $c_2 < \hat{c}_2$, follow the procedure in (2), exchanging between 1 and 0.
